# Perception of success among adults practicing various fighting arts

**DOI:** 10.3389/fpsyg.2024.1441706

**Published:** 2024-09-27

**Authors:** Tadeusz Ambroży, Natalia Serafin, Przemysław Pawelec, Paweł Adam Piepiora, Zbigniew Borysiuk, Bogdan Kindzer, Krzysztof Kasicki, Łukasz Rydzik, Wojciech J. Cynarski

**Affiliations:** ^1^Institute of Sports Sciences, University of Physical Education, Kraków, Poland; ^2^Faculty of Physical Education and Sport, Institute of Social Sciences, University of Physical Education in Krakow, Kraków, Poland; ^3^Institute of Physical Culture Studies, College of Medical Sciences, University of Rzeszow, Rzeszów, Poland; ^4^Faculty of Physical Education and Sports, Wroclaw University of Health and Sport Sciences, Wrocław, Poland; ^5^Faculty of Physical Education and Physiotherapy, Opole University of Technology, Opole, Poland; ^6^Faculty of Physical Education and Sport, Lviv State University of Physical Culture Named After Ivan Boberskyj, Lviv, Ukraine; ^7^Faculty of Medicine and Health Sciences, Andrzej Frycz-Modrzejewski Kraków University, Kraków, Poland

**Keywords:** martial arts, combat sport, success, perceptions of success, goal orientation

## Abstract

**Problem statement:**

Goal orientations, which influence learning and motivation, are categorized into task orientation (focused on skill development and personal mastery) and ego orientation (focused on outperforming others and gaining recognition). This study aims to explore how different demographics perceive success in martial arts.

**Approach and purpose:**

The research questions focused on identifying correlations between perceptions of success and demographic factors: (1) sex, (2) age, (3) type of martial art practiced, and (4) nationality. These factors were examined to understand their impact on success perceptions among martial arts practitioners.

**Materials and methods:**

The study utilized the Perception of Success Questionnaire (POSQ) to gather data from 268 participants—142 from Poland and 126 from Ukraine—selected through voluntary participation. Statistical analyses included standard deviation, coefficient of variability, Spearman’s rank correlation, and ANOVA for multifactor analysis.

**Results:**

The study revealed a weak correlation between the type of martial art practiced and perception of success (*r*_s_ = 0.38), indicating that the style of martial art has limited influence on success perception. There was a weak correlation between nationality and perception of success (*r*_s_ = 0.20), suggesting cultural factors do not play a role. A strong correlation was found between age and perception of success (*r*_s_ = 0.80), highlighting how perceptions evolve with age. A significant correlation was also found between sex and perception of success (*r*_s_ = 0.90), which may reflect broader cultural patterns influenced by globalization.

**Conclusion:**

While the study identifies important demographic correlations affecting perceptions of success in martial arts, the findings are constrained by the sample size and demographic distribution, which limits the generalizability of the results. Further research with a larger and more diverse sample is recommended to deepen understanding.

## 1 Introduction

Goal orientations are of two types: task and ego. Both are parts or manifestations of the motivational processes that affect learning: having a task-oriented goal, units are focused on developing new skills, attempting to understand their practice, raising the level of competence, and achieving a sense of mastery. In contrast, the central goal of ego is to focus on one’s own abilities and sense of self-worth (…) On the other hand, ego orientation has been linked to external motives for participation, such as social recognition and elevating one’s social status (De Andrade et al., 2008). Perceptions of success and task orientation are important issues in the psychology of sports, coaching, pedagogy, and physical education (Ames, 1992; Cynarski, 2016; Nicholls, 1984; Tenenbaum and Eklund, 2007). Goal orientation is an important component of personal motivational profiles in sports (Murcia et al., 2007; Tello et al., 2010; Meyer and Bittmann, 2018). When defining the task-oriented goal dimension, it can be assumed that it focuses on improving one’s own skills, acquiring knowledge, and the belief that, to be successful, one must put in the effort, try to understand the tasks, and cooperate with peers. Moreover, the ego-oriented dimension is characterized by the desire to surpass others and the belief that success requires greater abilities in relation to an external criterion. Although both dispositional goal orientations are characterized by a certain stability, they are not perceived as traits but as cognitive schemas subject to change (Morales-Sánchez et al., 2022).

The literature indicates that success in martial arts can be perceived through both task and ego orientations. Task orientation in martial arts focuses on the continuous improvement of techniques, self-development, and achieving personal mastery, which aligns with the traditional approach to these disciplines (Bowman, 2019). On the other hand, ego orientation may be observed in individuals who perceive success mainly through the lens of competition, defeating others, and gaining recognition, which may be more characteristic of contact sports such as MMA or boxing (Vertonghen et al., 2014).

Some authors have focused on the philosophy of traditional martial arts and ways to explain their impact on perceptions of success and aspirations (Bäck and Kim, 1979; Kim and Bäck, 2000; Cynarski, 2013, 2019; Zeng et al., 2013; Cynarski and Lee-Barron, 2014). The cultural conditions of martial arts perception and the internalization of the special ethos are indicated quite often (Dykhuizen, 2000; Zeng et al., 2013).

Understanding how different demographic groups perceive success in martial arts is crucial for tailoring training methods and personal development programs to the needs of participants. The study conducted by Cynarski et al. (2018) indicates that goal orientations can influence how martial arts practitioners engage in training and develop their skills. Therefore, linking the literature on task and ego orientations with the context of martial arts helps to better understand which factors can shape the perception of success in these disciplines, thus justifying the need for this study.

The analyses revealed that respondents’ goal orientation influenced their positive work-related behaviors, such as acceptance of the requirement to make an effort (Tuckey et al., 2002). Lochbaum et al. (2016), analyzing the knowledge obtained so far from various scientific studies, indicate the validity of the concepts relating to task orientation with correlated (at a significant level) adaptive achievements of people tested using the POSQ test. Therefore, formulating hypotheses relating to respondents involved in training in various forms of fighting arts is justified. The training processes related to this include (by assumption) the development of, among others, positive emotions and motives for improving and enriching skills, not only technical and tactical (Lochbaum et al., 2016).

Adopting this perspective allows for a more comprehensive approach to analyzing success in martial arts, taking into account both traditional values and the contemporary demands of sports competition. Thus, this study aims to understand how different goal orientations can influence the perception of success in a context that is deeply rooted in tradition while simultaneously evolving dynamically in the modern world.

The problem and aim of this study were to understand the “perception of success” among adults practicing various fighting arts. The study focused on the following research questions:

1. What is the correlation between the perception of success and the respondents’ sex?
2. What is the correlation between the perception of success and the respondents’ age?
3. What is the correlation between the perception of success and the respondents’ nationality?
4. What is the correlation between the perception of success and the type of fighting art practiced by the respondents?
5. How do sex, age, nationality, and type of fighting art collectively correlate with the perception of success among respondents?

By addressing these questions, the study aims to uncover the relationships between demographic factors and success perceptions, which can provide insights into how different groups define and pursue success in martial arts.

Based on the research questions, the following hypotheses were formulated:

1. There is a strong correlation between the respondents’ perception of success and their sex.
2. There is a strong correlation between the respondents’ perception of success and their age.
3. There is a strong correlation between the respondents’ perception of success and their nationality.
4. There is a strong correlation between the respondents’ perception of success and the type of fighting art they practice.
5. There is a strong correlation between the respondents’ perception of success and the combined factors of sex, age, nationality, and the type of fighting art practiced.

These hypotheses aim to explore how individual and combined demographic factors influence success perceptions among martial arts practitioners.

## 2 Materials and methods

The Perception of Success Questionnaire (POSQ) tool was used for the study, which was found to be a valid and reliable research tool. It is considered useful for both scientific and diagnostic purposes (Tomczak et al., 2020). The respondents used the Polish and Ukrainian versions of the questionnaire, respectively.

This tool has been used in similar studies on adults and young people who practice and play sports (Beck et al., 2013; Gómez-López et al., 2013; Noomen et al., 2011; Lochbaum et al., 2016; Cynarski et al., 2018). In recreational sports, in general, the aim is good “physical health, mental well-being, improvement of fitness level, and independence—achieving personal challenges and acquiring a definite position—all of which resemble a task orientation. In turn, the motives for participation in sports activities resemble ego orientation” (Beck et al., 2013). According to Martín et al. (2006), “task orientation is correlated with motivation and effort to achieve success, with sports practice enjoyment, anxiety over stressful situations, and a lower learning commitment and dedication” (Martín et al., 2006). However, fighting arts, and particularly traditional martial arts, have their own characteristics.

The number of respondents from Poland and Ukraine practicing various types of fighting arts chosen randomly with their voluntary participation amounted to *N* = 268 (142 and 126, respectively). These included individuals training in martial arts and combat sports in organized groups within associations and sports clubs.

A compilation and calculation of ranks were made based on the standard deviation, coefficient of variability, and strength of interdependence between variables using Spearman’s rank correlation and an ANOVA test for multivariate analysis. The significance level was set at *p* < 0.05. Data were analyzed using the Statistica software (Version 13; StatSoft, Palo Alto, CA, USA).

## 3 Results

Results were obtained based on the applied data collection method and are presented in the tables provided. The study was conducted from October 2022 to April 2023 in Poland (Rzeszów and Wrocław) and Ukraine (Lviv). The respondents provided anonymous responses by completing the POSQ test without a set time limit. The percentage participation of women and men, divided by country, is presented in Table 1.

**Table 1:** Data regarding examined individuals.

	Poland		Ukraine		Total	
Gender	*N*	%	*N*	%	*N*	%
Women	64	45.1	30	37.8	94	35
Men	78	54.9	96	62.2	174	65
Total	142	100.0	126	100.0	268	100

Owing to the difference in the number of respondents between Poland (142 individuals) and Ukraine (126 individuals), certain relationships between the number of respondents were observed. Polish women accounted for 45.1% (64) of the sample, compared to 54.9% of Polish men (78). For respondents from Ukraine, these proportions were 37.8% (30) women and 62.2% (96) men. Overall, 35% (94) of the patients were women, and 65% (174) were men. Variations among the surveyed respondents were observed based on age data. The results are summarized in Table 2.

**Table 2:** Average age of respondents by gender

	Poland	Ukraine	Overall average
Women	21,9	25,4	23,65
Men	21,3	25,9	23,6
Total	21,6	25,65	23,63

The average ages of the respondents (women and men) from both countries varied. The highest average score was recorded among individuals practicing martial arts and combat sports in Ukraine (women: 25.4 years, men: 25.9 years). The following were Polish women (21.9 years) and men (23.3 years). No statistically significant difference was found between the sexes (women: 23.65 years, men: 23.6 years).

This study utilized a diagnostic survey employing the POSQ toll. The questionnaire consisted of 12 affirmative statements (in a response form) and a scale for potential acceptance or rejection. These statements expressed the following attitudes: (1) I beat other people; (2) I am clearly superior; (3) I am the best; (4) I work hard; (5) I show clear personal improvement; (6) I outperform my opponents; (7) I reach a goal; (8) I overcome difficulties; (9) I reach personal goals; (10) I win; (11) I show other people I am the best; (12) I perform to the best of my abilities.

The surveyed individuals evaluated their attitudes toward the statements based on a scale ranging from A (strongly agree) to C (neutral) to E (strongly disagree). Each response was assigned a corresponding rank (1–5). It should be noted that questions 4, 5, 7–9, and 12 were categorized as task orientation, while questions 1–3, 6, 10, and 11 were categorized as ego orientation (Roberts et al., 1998). Hence, the total points awarded by the respondents to each response for each category ranged from 6 to 30. The results were used to analyze the acceptance or rejection of the above attitudes and any correlations or lack thereof. The first hypothesis concerned the strong correlations between respondents’ perceptions of success and sex. The results obtained during hypothesis testing are summarized in Table 3.

**Table 3:** Sums of points and statistics of responses regarding perception of success and gender of respondents.

	Women				Men			
	Sum of points answers	x̅	Sd	*V*	Sum of points answers	x̅	Sd	*V*
Task orientation	293	1.63	0.20	13%	937	1.63	0.25	15%
Ego orientation	454	2.52	0.57	23%	1.474	2.56	0.43	17%

x̅, mean; Sd, standard deviation; V, coefficient of variation.

The sum of the points for answers concerning task orientation was 293 for women and 937 for men. There was also a difference in ego orientation (454 vs. 1,474). The average responses for task orientation according to ranks (1–5) for respondents by gender were both 1.6. However, the average scores for ego orientation were 2.52 and 2.56, respectively.

The standard deviation for task orientation among women was 0.20, and that among men was 0.25. Whereas for ego orientation, the standard deviation values were 0.57 and 0.43, respectively. The coefficients of variability (expressed as percentages) for task orientation (13% for women and 15% for men) and ego orientation (23% for women and 17% for men) indicated low variability. In all cases, the variability in characteristics was not statistically significant.

The result of Spearman’s rank correlation test was 0.90. This indicates a fairly strong relationship between gender and the perception of success. Therefore, the hypothesis stating the presence of a strong correlation should be accepted.

The second hypothesis concerned the strong correlations between respondents’ perceptions of success and age. The results obtained during hypothesis testing are summarized in Table 4.

**Table 4:** Sum of points and statistics of responses regarding perception of success and age of respondents.

	Under 21 years old				21 years old and above			
	Sum of points answers	x̅	Sd	*V*	Sum of points answers	x̅	Sd	*V*
Task orientation	1.379	1.82	0.26	14%	1.553	1.73	0.33	19%
Ego orientation	1973	2.55	0.38	15%	2.220	2.59	0.47	18%

x̅, mean; SD, standard deviation; V, coefficient of variation.

The sum of the points for answers concerning task orientation among individuals aged up to 21 years was 1,379, and for those aged 21 years and older, it was 1,553. There was also a difference in ego orientation (1973 vs. 2,220). The average responses for task orientation according to ranks (1–5) for respondents aged up to 21 years were 1.82, and for those aged 21 years and above, it was 1.73. However, the average scores for ego orientation were 2.55 and 2.59, respectively. The standard deviation for task orientation among individuals aged up to 21 years was 0.38, whereas that among those aged 21 years and above was 0.33. Whereas for ego orientation, the standard deviation values were 0.38 and 0.47, respectively. The coefficient of variability values (expressed as percentages) for task orientation (14% for individuals aged up to 21 years and 19% for those aged 21 years and older) and ego orientation (15% for individuals aged up to 21 years and 18% for those aged 21 years and older) indicated low variability. In all cases, the variability in characteristics was not statistically significant. The results of the Spearman’s rank correlation test were 0.80. This indicates a strong relationship between respondents’ age and their perception of success. Therefore, the hypothesis stating the presence of a strong correlation should be accepted. The third hypothesis focused on the assumption that differences in the responses of respondents from Poland and Ukraine regarding the perception of success arose mainly from the nationality of the individuals participating in the study. The results are summarized in Table 5.

**Table 5:** Sums of points and statistics of responses regarding the perception of success and the nationality of respondents

	Poles				Ukrainians			
	Sum of points answers	x̅	Sd	*V*	Sum of points answers	x̅	Sd	*V*
Task orientation	1702	2.00	0.11	7%	1,230	1.63	0.23	14%
Ego orientation	2.259	2.67	0.29	19%	1928	2.55	0.45	18%

x̅, mean; SD, standard deviation; V, coefficient of variation.

The sum of the points for answers concerning task orientation among the Poles was 1702, and among the Ukrainians, it was 1,230. There was also a difference in ego orientation (2,259 compared to 1928). The average responses for task orientation according to ranks (1–5) for respondents by nationality were 2.00 Poles and 1.63 Ukrainians. However, the average scores for ego orientation were 2.67 and 2.55, respectively. The standard deviation for task orientation among the Poles was 0.11, and among the Ukrainians, it was 0.23. Whereas for ego orientation, the standard deviation values were 0.29 and 0.45, respectively. The coefficient of variability values (expressed as percentages) for task orientation (7% for Poles and 14% for Ukrainians) and ego orientation (19% for Poles and 18% for Ukrainians) indicated low variability. In all cases, the variability in characteristics was not statistically significant. The result of Spearman’s rank correlation test was 0.20. This indicates a weak relationship between respondents’ nationality and their perception of success. Therefore, the hypothesis that a strong correlation exists should be rejected. For the fourth hypothesis, it was assumed that martial arts and combat sports would be divided according to contact intensity. The “combat sports” category included MMA and boxing, while “martial arts” included judo and karate. The results are summarized in Table 6.

**Table 6:** Sum of points and statistics of responses regarding perception of success and type of fighting arts practiced by respondents

	Combat sports				Martial arts			
	Sum of points answers	x̅	Sd	*V*	Sum of points answers	x̅	Sd	*V*
Task orientation	720	1.08	0.40	37%	520	1.44	0.15	10%
Ego orientation	1.056	2.67	0.42	16%	872	2.42	0.53	22%

x̅, mean; SD, standard deviation; V, coefficient of variation.

The sum of the points for answers concerning task orientation among individuals practicing combat sports was 720, and for those practicing martial arts, it was 520. There was also a difference in ego orientation (1,056 vs. 872). The average responses for task orientation according to ranks (1–5) for respondents practicing combat sports were 1.08, and for those practicing martial arts, it was 1.44. Therefore, the difference was not statistically significant. However, for ego orientation, the averages were 2.67 and 2.42, respectively. The standard deviation for task orientation among individuals practicing combat sports was 0.40, and for martial arts, it was 0.15. Whereas for ego orientation, the standard deviation values were 0.42 and 0.53, respectively. The coefficient of variability values (expressed as percentages) for ego orientation (10, 22%) indicated low variability, while for task orientation (16, 37%) it indicated moderate and low variability. In all cases, the variability in characteristics was statistically significant. The result of Spearman’s rank correlation test was 0.38. This indicates a weak relationship between the type of fighting art practiced and the perception of success. Therefore, the hypothesis that a strong correlation exists should be rejected. The fifth hypothesis concerned a strong correlation between respondents’ perceptions of success and sex, age, nationality, and the type of fighting art practice. Martial arts and combat sports were divided according to contact intensity. “Combat sports” included MMA and boxing, while “martial arts” included judo and karate. The results obtained from the ANOVA tests are presented in Table 7.

**Table 7:** ANOVA test of perception of success by gender, age, type of practiced fighting arts, and nationality.

Variable	SS effect	df effect	MS effect	SS error	df error	MS error	*F*	*p*-value
Task orientation	27.55887	26	1.059957	84.4631	241	0.350469	3.024392	0.000004
Ego orientation	42.16512	26	1.621735	107.0939	241	0.444373	3.649491	0.000000

SS, sum of squares; df, degrees of freedom; MS, mean squares.

The *p*-values indicate that for both task orientation and ego orientation, the hypothesis stating a strong correlation between these variables and gender, age, type of fighting arts practiced, and nationality should be rejected. Therefore, no further tests were performed. However, to determine which of the analyzed means differed significantly from each other, *post hoc* tests were performed using Tukey’s honest significant difference (HSD) test. The application of this method revealed that the means were influenced by the responses provided by a 22-year-old female respondent from Ukraine practicing judo.

## 4 Discussion

Judo is both a martial art contributing to the group of Japanese “ways of martial arts,” known as budō, and a combat sport featured in the Olympic Games. According to research by Malchrowicz-Mośko et al., “motivation turned out to be the highest for recreational judokas who have been training for less than 10 years. People who remained motivated to practice judo at a competitive level for over 10 years highly appreciated aspects such as intrinsic motivation to experience stimulation, intrinsic motivation to know, and intrinsic motivation to accomplish” (Malchrowicz-Mośko et al., 2020).

Wolska et al. compared the motivations of judo and Brazilian jiu-jitsu athletes. It was found that “(1) higher total motivation for sports training in the judo group than in the Brazilian jiu-jitsu group suggests a positive impact of judo being part of the Olympic sports group. (2) A similar course of motivational profile lines and identical dominant components of motivation indicates an identical course of the motivation process for practicing both judo and Brazilian jiu-jitsu, which runs at different levels. (3) Both Judo and Brazilian jiu-jitsu training give people a sense of good health, physical fitness, and independence.” However, in both cases, athletes were involved in sports competitions, so they were simultaneously oriented toward achieving success in sports (the role of motivation in both orientations is presented in the Introduction) (Wolska et al., 2019).

The results indicating correlations between perceptions of success and factors such as age and nationality suggest that different life stages and cultural backgrounds can significantly influence how individuals define success. Older participants may place greater importance on self-improvement and mastery, which aligns with the traditional values of martial arts, as shown in previous studies (King and Williams, 1997). In younger age groups, which are more competition-oriented, success may be perceived through the lens of victories and external achievements. These differences may also result from changing life priorities and personal experiences (Nicholls, 1984). Similarly, differences in nationality may reflect diverse cultural approaches to success and competition, which is significant in the context of globalization and the intermingling of cultures. For example, in collectivist cultures, success may be more defined by contributions to the group rather than individual achievements (Markus, 1991).

Sometimes, the variable differentiating motivation is the type of fighting art practiced, while at other times, it is the cultural context, as in the case of research comparing karate practitioners from Europe and Japan (Meyer and Bittmann, 2018). Gender differences are often observed in various sports disciplines. The results of our own research (as presented above) generally align with similar studies, highlighting factors influencing individuals engaged in martial arts training (Cynarski et al., 2018; King and Williams, 1997; Vertonghen and Theeboom, 2012; Zeng et al., 2015). However, these studies provide new insights into the perception of success among adults practicing martial arts. Strong correlations between this perception and gender variables suggest significant cultural pattern unification in Western countries in the era of globalization. Additionally, no significant differences were found between those practicing martial arts and those training in combat sports, which differs from the results of earlier studies (Witkowski et al., 2013; Vertonghen and Theeboom, 2012). Ames (1992) research on the impact of task orientation on long-term engagement in sports indicates that individuals with a high task orientation tend to derive greater satisfaction from participation, which can also be applicable to martial arts. In contrast, studies conducted by Lochbaum et al. (2016) suggest that ego orientation is more associated with the level of competition and pressure to achieve.

In the study by Vertonghen et al. social diversity was found regarding the practiced types of martial arts and combat sports. However, the POSQ tool was only administered to adolescents aged 11–18 years (*n* = 33) (Vertonghen et al., 2014). A correlation between aggressiveness and goal orientation has often been observed in more intense contact-oriented combat sports. Significantly, “more immigrants were found among kick/Thai boxers” (Vertonghen et al., 2014; King and Williams, 1997; Vertonghen and Theeboom, 2012; Witkowski et al., 2013).

Although the results suggest a certain cultural pattern in the perception of success, caution should be exercised when making generalizations. The results may be specific to the studied group and may not reflect broader global trends. Cultural and contextual diversity can influence how success is defined and understood. It is recommended that future research cover a wider cultural spectrum to better understand these relationships. For instance, studies on differences in perceptions of success between individualistic and collectivist cultures could provide valuable insights (Triandis, 1996).

This issue requires further research on a larger scale within communities practicing various forms of martial arts, combat sports, and related systems (self-defense and combat).

### 4.1 Limitations of the study

While the authors acknowledge the limitations related to sample size and demographic representation, a more detailed analysis could help understand how these factors affect the results. A small sample size and lack of diversity in the representation of different demographic groups may limit the ability to generalize the findings. For example, the results may be more representative of specific age or cultural groups and may not apply to the broader population practicing martial arts. Therefore, future research should aim to increase participant diversity, allowing for a more comprehensive analysis and better understanding of how various demographic factors influence perceptions of success.

## 5 Conclusion

The study provides valuable insights into the impact of demographic factors on the perception of success in martial arts. Age and gender of participants are key determinants of how they perceive success, which is important for both practitioners and researchers. Understanding these relationships can aid in better designing training programs and motivational strategies that address the specific needs of different participant groups.

Future research should explore how specific martial arts styles influence the perception of success, considering a broader representation of various styles and cultures. Additionally, studies should include larger and more diverse samples to allow for better generalization of the results. Qualitative methods, such as interviews, would also be beneficial to gain a deeper understanding of participants’ motivations and attitudes towards success.

### 5.1 Practical implications

Martial arts instructors and sports psychologists should consider the age and gender of participants when designing training programs and motivational strategies. Age and gender can influence how participants perceive success and what motivates them to train. Therefore, it is important to tailor training and communication methods to better meet the needs of different demographic groups, which can enhance their engagement and satisfaction with the training. For example, younger individuals might be more focused on achievements and competition, whereas older individuals might value personal development and skill improvement more.

## Data Availability

The original contributions presented in the study are included in the article/supplementary material, further inquiries can be directed to the corresponding authors.

## References

[ref1] Ames C. (1992). Classrooms: goals, structures, and student motivation. J. Educ. Psychol. 84, 261–271. doi: 10.1037/0022-0663.84.3.261

[ref2] Bäck A. Kim D. (1979). Towards a Western philosophy of the eastern martial arts. J. Philos. Sport 6, 19–28. doi: 10.1080/00948705.1979.10654147

[ref3] Beck O. Monastyrska E. Wojciechowska M. Szrajda J. (2013). Achievement goals in exercise and sport from the public health perspective. Hygeia Public Healt 48, 505–508.

[ref1002] Bowman P. (2019). Deconstructing martial arts. Cardiff University Press. 182.

[ref4] Cynarski W. J. (2013). General reflections about the philosophy of martial arts. Ido Move. Cult. J. Martial Arts Anthrop. 1–6. doi: 10.14589/ido.13.3.1

[ref5] Cynarski W. J. (2019). Martial arts and combat sports. Towards the general theory of fighting arts Wojciech J. Cynarski. Katedra. doi: 10.18002/rama.v11i2s.4146

[ref6] Cynarski W. J. (2016). Towards the general theory of fighting arts. Revista de Artes Marciales Asiaticas. 11, 4–5.

[ref7] Cynarski W. J. Lee-Barron J. (2014). Philosophies of martial arts and their pedagogical consequences. Ido Movem. Culture. J Martial Arts Anthropol. 14, 11–19.

[ref8] Cynarski W. J. Pawelec P. Jong-Hoon Y. Vit M. Słopecki J. Kubala K. (2018). Perception of success among people Practising martial arts and combat sports. Central Eur. J. Sport Sci. Med. 21, 67–75. doi: 10.18276/cej.2018.1-08

[ref9] De Andrade A. Salguero A. Gonzalez-Boto R. Márquez S. (2008). The relationship of participation motivation to goal orientations and perceived physical ability in Brazilian swimmers. Psychologia 51, 157–169. doi: 10.2117/psysoc.2008.157

[ref10] Dykhuizen Jeffrey C. (2000). Culture, training, and perception of the martial arts: aikido example. J. Asian Martial Arts, 9.

[ref11] Gómez-López M. Antonio Granero-Gallegos J. Abraldes A. Rodríguez-Suárez N. (2013). Analysis of self-determined motivation in basketball players through goal orientations. Coll. Antropol. 37, 707–715, PMID: 24308207

[ref12] Kim D. Bäck A. (2000). The way to go: philosophy in martial arts practice. Korea (South): Nanam Publishing House.

[ref13] King L. A. Williams T. A. (1997). Goal orientation and performance in martial arts. J. Sport Behav.

[ref14] Lochbaum M. Çetinkalp Z. K. Graham K. A. Wright T. Zazo R. (2016). Task and Ego goal orientations in competitive sport: a quantitative review of the literature from 1989 to 2016. Kinesiology 48, 3–29. doi: 10.26582/k.48.1.14

[ref15] Malchrowicz-Mośko E. Zarebski P. Kwiatkowski G. (2020). What triggers us to be involved in martial arts? Relationships between motivations and gender, age and training experience. Sustainability 12:6567. doi: 10.3390/su12166567

[ref1003] Markus H. R. (1991). Cultural variation in the self-concept. The Self: Interdisciplinary Approaches/Springer.

[ref16] Martín J. J. S. Tendero G. R. Bañuelos F. S. (2006). Orientación y Clima Motivacional, Motivación de Logro, Atribución de Éxito y Diversión En Un Deporte Individual. Apunts 83, 5–11.

[ref17] Meyer M. Bittmann H. (2018). Why do people train martial arts? Participation motives of German and Japanese karateka. Societies 8:128. doi: 10.3390/soc8040128

[ref18] Morales-Sánchez V. Pérez-Romero N. Franquelo M. A. Balaguer I. Hernández-Mendo A. Reigal R. E. (2022). Task and Ego orientation in sport questionnaire (TEOSQ): psychometric properties in its digital version. Int. J. Environ. Res. Public Health. 19:172. doi: 10.3390/ijerph19063251, PMID: 35328939 PMC8954511

[ref19] Murcia J. A. M. Gimeno E. C. Coll D. G. C. (2007). Young athletes’ motivational profiles. J. Sports Sci. Med. 6:172. 24149326 PMC3786237

[ref20] Nicholls J. G. (1984). Achievement motivation: conceptions of ability, subjective experience, task choice, and performance. Psycholo. Rev. 91:328.

[ref21] Noomen G. Jbabli S. Barhoumi M. Barhoumi M. Sabeur H. Brahim A. (2011). Cross cultural validity of the Arabic version of perception of success questionnaire (children’ S version). Indian J. Appl. Res. 4, 468–471. doi: 10.15373/2249555X/JAN2014/145

[ref22] Roberts G. C. Treasure D. C. Balague G. (1998). Achievement goals in sport: the development and validation of the perception of success questionnaire. J. Sports Sci. 16, 337–347. doi: 10.1080/02640419808559362, PMID: 9663958

[ref23] Tello F. P. Holgado L. N. Martínez M. L. Calvo T. G. (2010). A structural model of goal orientation in sports: personal and contextual variables. Span. J. Psychol. 13, 257–266. doi: 10.1017/S1138741600003838, PMID: 20480694

[ref24] Tenenbaum G. Eklund R. C. (2007). Handbook of sport psychology. 3rd Edn. United States Inc: John Wiley & Sons.

[ref1004] Triandis H. C. (1996). The psychological measurement of cultural syndromes. American Psychol. 51: 407.

[ref25] Tomczak M. Walczak M. Kleka P. Walczak A. Bojkowski Ł. (2020). The measurement of goal orientation in sport: psychometric properties of the polish version of the perception of success questionnaire (Posq). Int. J. Environ. Res. Public Health 17:6641. doi: 10.3390/ijerph17186641, PMID: 32932998 PMC7558419

[ref26] Tuckey M. Brewer N. Williamson P. (2002). The influence of motives and goal orientation on feedback seeking. J. Occup. Organ. Psychol. 75, 195–216. doi: 10.1348/09631790260098677

[ref27] Vertonghen J. Theeboom M. (2012). Martial arts and youth: an analysis of contextual factors. Int. J. Adolesc. Youth 17, 237–241. doi: 10.1080/02673843.2012.687689

[ref28] Vertonghen J. Theeboom M. Pieter W. (2014). Mediating factors in martial arts and combat sports: an analysis of the type of martial art, characteristics, and social background of young participants. Percept. Mot. Skills 118, 41–61. doi: 10.2466/06.30.PMS.118k14w3, PMID: 24724512

[ref29] Witkowski K. Cynarski W. J. Błazejewski W. (2013). Motivations and determinants underlying the practice of martial arts and combat sports. Ido Movem. Culture. J. Martial Arts Anthrop. 1, 17–26. doi: 10.14589/ido.13.1.3

[ref30] Wolska B. Pujszo R. Janowska P. Wojdat M. Zając M. Pujszo M. (2019). The specificity of motivations in different combat sports and different lengths of the sports career. Baltic J. Health Physical Act. 11, 109–116. doi: 10.29359/BJHPA.11.3.11

[ref31] Zeng H. Z. Cynarski W. J. Xie L. (2013). “Martial arts anthropology, participants’ motivation and behaviours” in Martial arts in Chanshu: Participants’ motivation, practice times and health behaviours (Lambert Academic Publishing).

[ref1008] Zeng H. Z. Cynarski W. J. Xie L. (2015). Exploring motivations of taekwondo athletes/students in new york city. World J. Edu. 5, 51–63.

